# Role of Donor Unrestricted T Cells (DURTs) in TB Host Defense: Implications for Novel TB Vaccine Development

**DOI:** 10.3390/vaccines14040365

**Published:** 2026-04-21

**Authors:** Dylan Kain, David Michael Lewinsohn, Deborah Anne Lewinsohn

**Affiliations:** 1 University Health Network, Department of Medicine, University of Toronto, Toronto, ON M5S 1A1, Canada; dylan.kain@utoronto.ca; 2 Division of Pulmonary, Allergy, and Critical Care Medicine, Oregon Health and Sciences University, Portland, OR 97239-3098, USA; lewinsod@ohsu.edu

**Keywords:** tuberculosis, *Mycobacterium tuberculosis*, donor unrestricted T cells (DURTs)

## Abstract

Tuberculosis (TB) is the leading cause of infectious disease-related death globally. Most TB vaccine strategies have focused on conventional CD4 T cell responses, but to date, these have failed to deliver durable sterilizing protection. Donor unrestricted T cells (DURTs), including CD1-restricted T cells, HLA-E-restricted T cells, MR1-restricted T cells and γδ T cells represent an attractive complementary target for future TB vaccine development. They recognize antigens through conserved, non-polymorphic restricting elements and are therefore broadly targetable across genetically diverse populations. They are also enriched at mucosal sites, have rapid effector and cytotoxic capacities and recognize conserved mycobacterial ligands. Emerging human and animal data support their participation in antimycobacterial immunity and suggest they can be shaped by BCG vaccination and other immunization strategies. Here, we review the evidence for DURT involvement in TB host defense, assess their strengths and current limitations as vaccine targets, and discuss how DURT-directed approaches may help to enable faster, broader, and more durable protection against *Mycobacterium tuberculosis*.

## 1. Introduction

With 1.25 million deaths in 2023, tuberculosis (TB), caused by infection with *Mycobacterium tuberculosis* (Mtb), is the leading cause of infectious disease-related mortality worldwide [1]. Despite this, the only licensed vaccine for TB, the bacille Calmette–Guérin (BCG), is over 100 years old, and while it provides some benefit against severe and disseminated TB in early life, it has not shown consistent protection in adults [2]. Novel efficacious vaccines are urgently needed.

Mtb is spread via aerosolized droplets released from contagious individuals that are inhaled into the respiratory tract and lungs [3]. There, the bacilli are phagocytosed by front-line immune cells, including alveolar macrophages and airway epithelial cells [3]. However, digestion is generally avoided by virulence factors that prevent phagolysosome fusion and acidification [3]. Once inside macrophages, Mtb can persist in a niche that allows for bacterial survival and replication, while simultaneously driving inflammation that leads to granuloma formation, a hallmark of TB pathology [3]. The immune response that controls, but often fails to sterilize, Mtb is highly dependent on T cells [3]. T lymphocytes provide cytokines such as IFN-γ and TNF that activate infected macrophages, enhance antimicrobial effector mechanisms, and help to contain bacilli within granulomas [3]. When contained, this is termed as an Mtb infection (TBI). Among T lymphocytes that control Mtb infection, studies in well-established models—including aerosol Mtb infection in mice (e.g., C57BL/6 models) [4] and NHP models such as Mtb-infected rhesus and cynomolgus macaques [5]—demonstrate that conventional CD4^+^ T cells, through the recognition of peptides presented by Major Histocompatibility Complex (MHC) Class II molecules, play a particularly crucial role in protection against TB. In particular, CD4 T cells have been shown to play an important role in bacterial control and dissemination [6]. The importance of CD4 T cells in humans has been starkly highlighted by the HIV epidemic, where the loss of CD4 T cells was associated with profound susceptibility to TB disease and reactivation, making TB the leading cause of death in people living with HIV [7]. Therefore, for decades, CD4 T cells have been positioned at the center of TB vaccine design.

Yet, mounting evidence demonstrates that CD4-focused strategies may be insufficient in isolation to achieve sterilizing or durable protection. Multiple studies have shown that the magnitude of IFN-γ production by CD4 T cells—a widely used surrogate of protective immunity—does not correlate with protection against TB [8]. Perhaps the most sobering example was the results of a Phase IIb trial of South African infants comparing BCG to BCG boosted with the MVA85A vaccine, a viral vectored vaccine based on Modified Vaccinia Ankara, expressing antigen 85A from Mtb [9]. In this trial, despite eliciting modest but measurable CD4 T cell responses, the MVA85A boost provided no additional protection to BCG alone. Equally telling is the epidemiological observation that prior TB, which leaves behind substantial populations of antigen-experienced CD4 T cells, does not confer reliable immunity against reinfection [10]. These data suggest that IFN-γ-producing CD4 T cells are necessary but not sufficient for effective host defense, and that correlates of protection remain elusive.

Collectively, these shortcomings demand a broader view of TB immunity. While CD4 T cells remain indispensable, they are unlikely to act alone. Vaccine development must therefore consider additional arms of the immune system that could complement or even surpass conventional pathways. CD8 T cells have been implicated in direct antimycobacterial activity through cytotoxicity, cytokine production, and the recognition of infected cells [11]. In parallel, a growing body of work supports roles for B cells and antibodies in TB, including opsonization, modulation of myeloid responses, and organization of lung granulomas [12], while innate populations such as NK cells and macrophages have been shown to be trained through previous exposure to BCG, leading to enhanced Mtb killing and protection [13,14]. Together, these observations underscore that effective TB vaccines may need to engage coordinated adaptive and innate programs beyond classical CD4 T cell immunity.

Donor unrestricted T cells (DURTs) have emerged as a particularly appealing class of lymphocytes with unique potential for TB vaccine development. Unlike conventional T cells, which recognize peptides presented by highly polymorphic classical MHC molecules, DURTs recognize antigens presented by non-polymorphic or monomorphic molecules. They are termed as donor unrestricted, because these restricting elements are highly conserved and shared across nearly all individuals, meaning the same antigen–receptor interactions can, in principle, be engaged in any donor, independent of the Human Leukocyte antigen (HLA) background. This key feature circumvents one challenge of the T cell vaccine design: the extensive HLA diversity across human populations that complicates universal vaccine efficacy [15]. Moreover, eliciting DURTs as well as conventional T cells likely broadens the T cell response beyond targeting peptides to targeting non-peptide antigens produced by Mtb.

**Figure 1:**
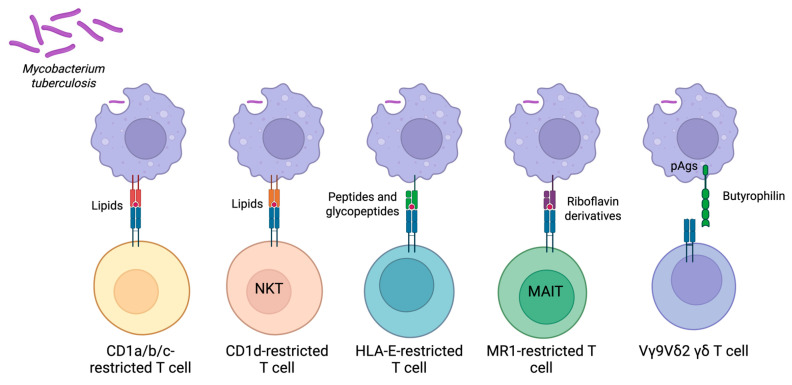
Summary of major types of DURTs. CD1a/b/c-restricted T cells and NKT cells (CD1d-restricted T cells) recognize lipid antigens presented from CD1. HLA-E-restricted T cells recognize peptides and glycopeptides presented by HLA-E. MR1-restricted T cells recognize riboflavin derivatives presented via MR1. Vγ9Vδ2 γδ T cells recognize butrophylin molecules after they undergo a conformational change induced by phosphoantigens (pAgs). Vδ1 and other non-Vγ9Vδ2 γδ T cells are not depicted in the full manner in which they are processed and recognizing antigens remains an ongoing area of research.

The DURT family encompasses several distinct subsets (Figure 1):

• CD1-restricted T cells include invariant natural killer T (iNKT) cells restricted by CD1d, as well as T cells restricted by CD1a, CD1b, or CD1c molecules.
• HLA-E-restricted T cells recognize non-classical peptides and glycopeptides and can modulate immunity at mucosal barriers.
• MR1-restricted T (MR1T) cells, a subset of which are commonly termed MAIT cells, detect riboflavin-derived metabolites conserved across many microbes, including Mtb.
• γδ T cells sense diverse stress-induced ligands and non-peptide metabolites and can mount rapid effector responses during infection.

Several unifying features make DURTs attractive targets for TB vaccine design. First, they are enriched at mucosal surfaces, particularly the respiratory tract, positioning them at the frontline of the Mtb encounter [16]. Second, they are primed for rapid activation, enabling them to deliver immediate effector functions—including cytotoxicity and pro-inflammatory cytokine production—that could constrain early bacillary replication [15,17,18,19]. Indeed, γδ T cells demonstrate increased cytotoxicity following BCG vaccination [20], supporting their responsiveness to vaccine-induced priming. Third, the antigens recognized by DURTs—lipids, metabolites, and non-peptide ligands—are often highly conserved across microbial species and less prone to antigenic variation than classical peptides [15]. This reduces the likelihood of immune escape and opens the possibility of broadly protective vaccines.

Finally, DURTs operate at the interface of innate and adaptive immunity. Their innate-like rapidity allows them to amplify early responses and shape the inflammatory milieu, while their adaptive features (such as memory formation and TCR clonotypic selection) enable longer-term immune imprinting [15]. Through feedback on innate cells such as macrophages, DURTs can enhance microbial clearance and polarize responses in directions that are favorable for host defense [15,19].

The appeal of DURTs is not merely theoretical. Across their diverse subsets, DURTs are consistently enriched and activated in the lungs of TB patients, suggesting a shared evolutionary role in host defense [16]. Experimental models also underscore their importance. The critical role of CD8α T cells, for example, has been demonstrated in both protective immunity against TB and in achieving sterilizing immunity with intravenous BCG vaccination—one of the few experimental strategies to fully eradicate Mtb in animal models [6,21,22]. This was shown through selective depletion of CD8α, which will target all CD8^+^ T-cells, including CD8^+^ DURT populations, or CD8β depletion, which will primarily target conventional CD8^+^ T cells. With CD8β depletion, little difference was seen in Mtb progression and there was no loss of IV BCG mediated protection. Conversely, CD8α depletion led to severe worsening of TB disease and significant loss of IV BCG-mediated protection. Such findings highlight the possibility that combinations of DURTs, acting alongside conventional T cells, may be necessary to achieve sterilizing immunity in humans.

While DURT subsets are often studied in isolation, it is likely that they function as part of a coordinated immune network, rather than as independent effectors. These populations share key features—including rapid effector function, recognition of conserved microbial ligands, and enrichment at mucosal sites—but differ in their antigen specificity, tissue distribution, and kinetics of activation. It remains unclear whether DURT subsets act redundantly, synergistically, or at distinct stages of infection, and the current data are insufficient to definitively rank their relative importance for protection. In one study or a person who lacked MR1T cells, due to an MR1 genetic mutation, there was an apparent increase in γδ T cells [23], suggesting that they possibly serve in a redundant manner, but this is largely unproven. Addressing these questions will be essential for rational vaccine design and may require integrated approaches that engage multiple DURT compartments simultaneously.

Together, these insights suggest that the future of TB vaccine design may rest on moving beyond conventional paradigms. By leveraging DURTs, vaccines could overcome the barriers that have hindered past efforts: HLA restriction, antigenic variability, and delayed adaptive responses. This review will explore the distinct DURT subsets, the evidence for their roles in TB host defense, and their potential as novel targets for vaccine development in detail. In doing so, it seeks to re-frame TB vaccine design not as a problem of optimizing CD4 responses, but as an opportunity to harness the full spectrum of immunological diversity shaped by human–mycobacterial co-evolution, including CD8 T cells, B cells, innate cells and DURTs.

## 2. CD1-Restricted T Cells in Mycobacterial Immunity

CD1-restricted T cells encompass a broad spectrum of T lymphocytes, including both αβ and γδ TCR-expressing subsets, that recognize lipid antigens presented by CD1 molecules. The CD1 family is divided into group 1 (CD1a, CD1b, CD1c), group 2 (CD1d), and group 3 (CD1e). While group 1 and 2 molecules present lipids to T cells, CD1e functions exclusively in intracellular processing within the endoplasmic reticulum. Given that approximately 40% of the Mtb cell wall and 6% of its genome are devoted to lipid metabolism [24], it is not surprising that lipid-specific T cell responses represent an important dimension of antimycobacterial immunity.

### 2.1. CD1a-Restricted T Cells

CD1a can present certain mycobacterial lipid antigens, including sulfated and lipopeptide molecules, which stimulate the production of protective cytokines such as TNF and IFN-γ. However, compared to other CD1-restricted subsets, CD1a-restricted T cells respond to Mtb at much lower frequencies. Furthermore, CD1a is most abundantly expressed on Langerhans cells in the skin—a site rarely infected by Mtb—whereas conventional dendritic cells (DCs) show much lower expression [25]. In the skin, CD1a-restricted T cells are often autoreactive, recognizing host-derived lipids generated during inflammation or stress. While some responsiveness to Mtb has been reported, these responses are weaker than those elicited by *Mycobacterium leprae* and are most robust when antigens are presented by skin-derived Langerhans cells, rather than monocyte-derived DCs [25].

CD1a-restricted T cells are defined by their response to CD1a^+^ antigen presenting cells (APCs) but not CD1-negative APCs, whose response is blocked by an anti-CD1a antibody. CD1a-restricted αβ T cell clones have previously been shown to recognize Mtb lipid fractions and this led to the discovery of didehydroxymycobactin, a lipopeptide intermediate in mycobactin biosynthesis, as a CD1a-presented antigen [26]. Importantly, Mtb-infected DCs, but not uninfected DCs, were able to present this antigen, confirming its natural processing and presentation during infection [26]. The most compelling evidence for CD1a-restricted T cell function arises from studies of a different mycobacterium, *M. leprae*, the causative agent of leprosy. Because *M. leprae* primarily targets the skin and peripheral nerves, it exploits the same niches where CD1a expression is highest. Leprosy patient-derived CD1a-restricted T cells respond strongly to lipopeptides and sulfated glycolipids, with Langerhans cells directly presenting *M. leprae* antigens to the infiltrating T cells. In tuberculoid leprosy, in the form of the disease characterized by effective cell-mediated immune control, CD1a-restricted T cell activity is enriched. From such lesions, αβTCR-expressing clones have been isolated and proliferated in response to CD1a+ DCs pulsed with *M. leprae* extracts, and they produced IFN-γ in a strictly CD1a-dependent manner [25].

### 2.2. CD1b-Restricted T Cells

Among group 1 molecules, CD1b-restricted T cells are the best characterized and provide some of the strongest evidence for protective roles in TB. Early work by Porcelli et al. (1992) demonstrated that CD1b-restricted T cell clones could lyse Mtb-infected, CD1b-expressing cells, but not those expressing CD1a or CD1c [27]. Subsequent studies identified several key lipid antigens, including mycolic acids and trehalose dimycolate, lipoarabinomannan, which activated CD1b-restricted T cells to both lyse antigen-pulsed monocytes and secrete IFN-γ [28,29]. Subsequently, glucose monomycolate (GMM) was discovered as a CD1b-presented antigen recognized by the LDN5 clone [30]. Additional lipid antigens include Ac2SGL, a sulfoglycolipid shown to restrict Mtb growth through IFN-γ and granulysin production by CD1b-restricted T cells. Strikingly, responses to Ac2SGL were detectable in individuals with TBI but not uninfected individuals, consistent with clonal expansion after Mtb exposure [31].

Other antigens include glycerol monomycolate (GroMM), which elicits IFN-γ responses in BCG-vaccinated individuals and individuals with TBI donors, but not in patients with active disease, pointing to a defect in antigen-specific memory during progression to TB disease [32]. Mycolic acid-specific CD1b-restricted T cells have also been found to be enriched at disease sites and persist post-treatment, though they are not robustly induced by BCG vaccination, potentially reflecting structural differences in lipids between BCG and Mtb. This observation highlights a potential avenue for rBCG design—engineering strains to express Mtb-like mycolates could promote durable CD1b-restricted memory and improved vaccine efficacy [33,34,35].

The development of CD1b tetramers revolutionized the field, enabling the direct visualization and characterization of GMM-specific T cells. Kasmar et al. (2011) and Van Rhijn et al. (2013) identified two major subsets: GEM T cells, defined by a conserved TRAV1-2–TRAJ9 α-chain, CD4 expression, and expansion in TB patients; and LDN5-like T cells, which use TRAV17/TRBV4-1 and show broader diversity in TCR and coreceptor usage [36,37,38]. Functionally, CD1b-restricted T cells that are specific for lipoarabinomannan from individuals with TBI have been shown to suppress Mtb growth in vitro in monocyte-derived DCs [39]. Although mice lack group 1 CD1 molecules, transgenic models such as human CD1b transgenic mice provide proof-of-principle: adoptive transfer of CD1b-restricted T cells reduced the Mtb burden, supporting a protective role in vivo [40].

### 2.3. CD1c-Restricted T Cells

CD1c is widely expressed across tissues, and its lipid antigens are recognized by both αβ and γδ T cells, reflecting an exceptionally diverse TCR repertoire [41,42,43,44,45,46,47,48]. CD1c-restricted T cells are defined by their response to CD1c^+^ APCs but not CD1-negative APCs, whose response is blocked by anti-CD1c antibody. The first evidence for a role in Mtb control came from Moody et al., who found that CD1c-restricted T cells from individuals with TBI proliferated in response to isoprenoid glycolipids resembling Mtb-derived lipids [47]. Following this, mannosyl-β1-phosphomycoketide (MPM) and phosphomycoketide (PM) were identified as natural CD1c-presented antigens [49]. More recently, to address stability limitations for vaccine use, Reijneveld et al. synthesized an MPM analog that was resistant to enzymatic degradation, which successfully expanded MPM-specific T cells, providing a path forward for harnessing CD1c ligands in vaccine strategies [50].

### 2.4. CD1d-Restricted NKT Cells

Group 2 CD1 is represented by CD1d, which presents lipids to natural killer T (NKT) cells, including invariant NKT (iNKT) cells and some Vδ1 γδ T cells [51,52]. The NKT subsets include type I iNKT cells, defined by a semi-invariant Vα24-Jα18 TCR [53,54], and type II NKT cells with more diverse repertoires [55]. Because CD1d is the only CD1 molecule expressed in mice, iNKT cells are the best characterized of the CD1-restricted populations. Discovery of α-galactosylceramide (α-GalCer), a high-affinity CD1d ligand, enabled the generation of tetramers and facilitated a detailed dissection of NKT cell biology in both mice and humans [56,57,58,59,60].

In 2004, Fischer et al. demonstrated that phosphatidylinositol mannoside (PIM) from BCG could stimulate murine iNKT cells via CD1d, driving IFN-γ production [61]. Additional studies have highlighted iNKT’s roles in TB control. For instance, Sada-Ovalle et al. reported that murine iNKT cells upregulated CD69 when exposed to Mtb-infected macrophages, and splenocytes from wild-type—but not iNKT-deficient—mice’s restricted bacterial growth in a CD1d-dependent manner [62]. Pure iNKT cell lines suppressed Mtb replication in macrophages, and the adoptive transfer of iNKT cells into irradiated, infected mice markedly reduced bacterial burdens in both the lungs and spleen [62]. However, not all studies are concordant; some report no difference in disease outcome between wild-type and CD1d-deficient mice, suggesting context-dependent or redundant protective mechanisms [63].

CD1-restricted T cells comprise a diverse set of lipid-reactive populations that contribute to antimycobacterial immunity in distinct ways. The evidence is strongest for CD1b-restricted T cells, which recognize multiple Mtb lipids [27,28,29,30,31,32] and exert antimicrobial functions [39,40], while CD1c-restricted T cells are emerging as important responders with broad TCR diversity [41,42,43,44,45,46,47,48] and translational potential through synthetic lipid analogs [49,50]. CD1a-restricted T cells appear to be more relevant to *M. leprae* than TB [25], whereas CD1d-restricted NKT cells show protective effects in some murine models [62]. Importantly, the limited induction of lipid-specific memory T cells by conventional BCG highlights an opportunity for recombinant BCG and lipid-based immunogens to enhance durable CD1-restricted T cell responses and strengthen vaccine-mediated protection [33,34,35]. However, much of the evidence for CD1-restricted T cell function in TB derives from in vitro studies, human association data, or transgenic animal models, and definitive evidence of their non-redundant protective role in human infection remains limited.

## 3. HLA-E-Restricted T Cells

In humans, three MHC class Ib molecules—HLA-E, HLA-F and HLA-G—have been described as potential antigen-presenting molecules for T cells. Among these, HLA-E is the best characterized, while the capacity of HLA-F and HLA-G to function as T cell restriction alleles remains largely speculative [64]. This review will focus on HLA-E-restricted T cells as a result.

HLA-E is structurally homologous to classical MHC class I (A/B/C) molecules and is composed of a heavy chain (α1–α3) with associated β_2_-microglobulin and can present peptides [65]. HLA-E is, however, functionally distinct in several critical ways. Unlike classical MHC molecules, which are highly polymorphic, HLA-E has limited polymorphism, with only two common alleles (HLA-E01:01 and HLA-E01:03), which differ by only a single amino acid. HLA-E binds a small subset of nonameric peptides, originally derived from the leader sequences (signal peptides) of HLA-A, -B, -C and -G molecules. HLA-E also serves a dual role in the cell, as it serves as both a ligand for innate receptors (especially on NK cells) and as a restriction element for αβ T cells. HLA-E-restricted T cells are defined by their recognition of HLA-E^+^ APCs and when they can be blocked by HLA-E-blocking antibodies, but not anti-HLA-A/B/C antibodies [66].

HLA-E was first described for its role as a ligand for innate receptors. Its surface expression with self-leader peptides reflects “cell health.” These surface HLA-E molecules are loaded with self-leader peptides, which are nine-amino-acid peptides originating from the leader sequences of classical HLA-I molecules, known as VL9 peptides. These loaded VL9-peptides can then be recognized by CD94/NKG2 receptor families on NK cells and some T cells, with NKG2A/B binding being inhibitory and NKG2C being activating. When HLA-E expression is lost or altered, such as in the setting of viral downregulation, cellular stress, transformation or others, this leads to a “missing-self” signal for NK cells and leads to their activation.

Although HLA-E was first described to present leader peptides to CD94/NKG2 receptor families, this is not its only role. In this regard, Heinzel et al. provided the first evidence of TCR-mediated recognition of a pathogen-derived antigen, demonstrating HLA-E-restricted recognition of Mtb-infected cells, by a CD8 T cell clone [67]. Harriff et al. subsequently defined the Mtb ligand recognized as a glycopeptide, a peptide derived from MPT32 that required N-terminal O-linked mannosylation by a mannosyltransferase encoded by the Rv1002c gene [68].

Beyond VL9 peptides, HLA-E can also present unmodified peptides derived from Mtb [66,69], as well as from a variety of other microbial pathogens, including Mtb, HIV, cytomegalovirus (CMV), *Salmonella typhimurium*, and Epstein–Barr virus [69,70,71]. Joosten *et al*. showed that in people sensitized to Mtb through BCG vaccination or Mtb exposure, there was HLA-E-restricted peptide presentation to CD8^+^ T cells, leading to their proliferation and activation [66]. These T cells also lysed Mtb-infected cells in an HLA-E-dependent manner. McMurtrey et al. further characterized the Mtb ligandome displayed by HLA-E, demonstrating 28 Mtb ligands from 13 different proteins that were displayed by HLA-E [69]. Subsequent work has characterized the frequency and phenotype of HLA-E-restricted T cells in humans with TBI. Using HLA-E tetramers loaded with defined Mtb peptides, both CD8^+^ and CD4^+^ HLA-E-restricted T cells have been detected in the peripheral blood of individuals with TB and asymptomatic TB infection (TBI), as well as in those co-infected with HIV [72]. These T cells display heterogeneous memory phenotypes—including central and effector memory subsets—and express activation and inhibitory receptors such as PD-1, KLRG1, and 2B4, which are comparable to bulk T-cell populations. The observation that these cells persist across different clinical states and co-infection backgrounds underscores that HLA-E-restricted responses are a stable component of the human anti-mycobacterial repertoire.

Analysis of the TCR repertoire of HLA-E/Mtb-specific CD8^+^ T cells demonstrate broad clonotypic diversity, rather than a stereotyped or invariant pattern, indicating that these responses are shaped by conventional, polyclonal TCR selection, rather than the semi-invariant biases that are characteristic of donor unrestricted T cells [73]. Functionally, these HLA-E-restricted T cells are capable of recognizing and killing Mtb-infected macrophages and restricting bacterial growth in vitro [74]. Notably, this cytolytic activity persists in the setting of HIV-1 co-infection, a context in which classical HLA-I expression is often downregulated, but HLA-E remains stable. This finding highlights HLA-E as a potentially advantageous target for vaccine strategies in populations with high HIV prevalence.

Mtb infection itself upregulates HLA-E expression on infected macrophages and DCs via IFN-γ–dependent and type I interferon pathways, resulting in the enhanced engagement of CD94/NKG2A on NK and CD8^+^ T cells [66,75]. While this may facilitate antigen presentation to HLA-E-restricted T cells, it can simultaneously promote inhibitory signaling that suppresses cytotoxic effector functions, mirroring viral immune-evasion mechanisms such as HCMV UL40-mediated stabilization of HLA-E. Thus, HLA-E expression during Mtb infection may serve as a double-edged sword, both enabling unconventional T-cell recognition and dampening NK/T-cell activation through NKG2A-mediated checkpoints.

In non-human primate models and human bronchoalveolar lavage (BAL) samples, HLA-E/Mtb-specific T cells are enriched in the lung compartment, where they likely participate in local immune surveillance [76]. However, their abundance has variably correlated with bacterial burden rather than protection, suggesting that while these cells are drawn to sites of infection, their functional contribution to host defense versus immunopathology remains to be established. The current data largely reflect an association with infection, rather than direct evidence of protection.

The limited polymorphism of HLA-E offers a rare opportunity for population-wide coverage, bypassing the allele-restriction barriers of classical MHC-I molecules. HLA-E-restricted responses can be primed by diverse vectors and may be especially valuable for mucosal or tissue-resident T-cell immunity—a key target for TB vaccines [73]. However, standard BCG vaccination does not appear to robustly expand these responses, indicating that vaccine strategies targeting HLA-E cells would need to include HLA-E-restricted antigens, along with tailored delivery systems to effectively promote HLA-E loading and presentation [66].

Overall, the growing body of evidence supports a model in which HLA-E acts as both an antigen-presenting molecule for unconventional CD8^+^ T cells and an immune checkpoint regulator. Harnessing the protective potential of this pathway—while mitigating its inhibitory signaling—represents an emerging frontier in donor unrestricted T cell-based vaccine design for TB.

## 4. MR1-Restricted T (MR1T) Cells

MR1T cells are highly conserved across mammalian species which may suggest a strong evolutionary pressure to maintain MR1 and their important role in immune protection [77]. MR1-restricted T (MR1T) cells are defined by their recognition of small molecules, including microbial, self, environmental metabolites, as well as drug and drug-like molecules [78,79,80,81] presented by the monomorphic MHC class 1-related molecule (MR1) [15]. MR1T cells were first described in 1993, as a common type of CD4-CD8-cell expressing an invariant TCR α-chain (TRAV1-2/TRAJ33) [54]. These TRAV1-2-expressing cells were later termed mucosal associated invariant T (MAIT) cells, given their invariant α-chain, enrichment in mucosal surfaces and later demonstration that they are restricted by the monomorphic MR1 molecule [82]. We now recognize that MAIT cells are only a subset, albeit a major subset, of the broader class of MR1-restricted T cells, which are generally defined through their binding to MR1-5-OP-RU-tetramers [83]. In adults, MAIT cells are the most common MR1T cell and are defined by their expression of an invariant TCR α-chain (TRAV1-2/TRAJ33/20/12 in humans), paired with a limited array of Vβ segments [83,84], and often express high levels of CD161 [85,86] and CD26 [87].

MR1T cells have been found to be important in protection against a wide array of microbes, including *Klebsiella pneumoniae* [88], *E. coli* [19], and mycobacterial pathogens [89]. Human and experimental data support a model in which MR1T cells act as early sensors and effectors during Mtb infection. MR1T cells are abundant in the lung and airways, enriched at mucosal surfaces, and are poised to respond rapidly to infected antigen-presenting cells [90,91,92]. Upon recognition of MR1-presented ligands, MR1T cells produce IFN-γ, TNF, GM-CSF, IL-17, and cytotoxic mediators such as granzymes and perforin, all of which are relevant to antimycobacterial immunity [90,91,92]. In vitro, human MR1T cells can restrict intracellular mycobacterial growth in infected macrophages in an MR1-dependent manner, through a combination of direct cytotoxicity and macrophage-activating cytokines [89]. BCG vaccination, which provides protection against TB in early life, has been shown to increase pro-survival and cytotoxic programs in MR1T cells, potentially priming them for protection against Mtb [93].

Multiple lines of evidence associate MR1T cells with protection-relevant immune states in TB. Cross-sectional studies in humans demonstrate that MR1T cells are reduced in the blood of those with TB, while being enriched in the lungs [91]. TB is often associated with peripheral depletion and functional exhaustion of MR1T cells, whereas, in some studies, individuals with TBI or controlled exposure retain MR1T cells with preserved cytokine and cytotoxic capacity [94,95]. These observations suggest either selective recruitment to tissues or preferential loss/dysfunction during progressive disease and are consistent with a protective role that is compromised in TB.

Further evidence for the importance of MR1T cells in protection from TB is through work by Seshadri et al., where in Vietnam, polymorphisms in MR1 that impaired MR1 gene expression were associated with significantly increased risk of meningeal TB [96]. In a group of individuals with very frequent Mtb exposures, but that resisted the development of TBI, as defined by a positive Interferon gamma release assay (IGRA) and/or TB skin test (TST), there was a significant increase in MR1T cell frequency, suggesting a possible role in protection from Mtb infection [97]. In non-human primate models, MR1T cells expand in the lung following Mtb challenge and acquire effector programs that are compatible with early containment of infection, strengthening the biological plausibility that MR1T cells participate in protective immunity [98,99].

Collectively, although definitive causal evidence in humans remains challenging, the convergence of antigen specificity, lung enrichment, rapid effector function, and association with controlled infection phenotypes supports a potential contribution of MR1T cells to host defense against TB, although to date, much of this evidence remains associative and causality in humans has not been definitively established. Evidence from murine models further supports a role for MR1-restricted T cells in antimycobacterial immunity [89]; however, the low frequency and divergent biology of MAIT cells in mice limit the extent to which these systems can fully capture the contribution of these cells in humans.

The donor-unrestricted nature of MR1, together with the semi-invariant TCR usage and rapid effector potential of MR1T cells, makes this compartment an attractive and underexplored target for TB vaccine development. Current TB vaccines, including BCG and most subunit candidates, are primarily designed to elicit classical CD4 T-cell responses, which, while necessary, have proven insufficient for durable protection. Incorporating MR1T biology into vaccine strategies could address several limitations of existing approaches.

First, MR1 ligands are small, chemically defined metabolites, raising the possibility of directly incorporating stable MR1 agonists into vaccine formulations. Synthetic MR1 ligands or pro-ligands could be used to selectively expand and program MR1T cells in vivo, analogous to invariant NKT-cell-based adjuvant strategies. Second, MR1T cells are naturally enriched at mucosal sites, suggesting that mucosal vaccination strategies could preferentially harness their protective potential in the lung. Third, MR1T cells may be particularly relevant in early life, when conventional T-cell immunity is immature but MR1T cells are rapidly shaped by microbial exposure, highlighting opportunities for neonatal or infant vaccine approaches [100,101,102,103].

The emerging data indicate that MR1T cells are not a uniform population, but encompass functionally distinct subsets, including cytotoxic, interferon-responsive, and tissue-resident programs. Future vaccines will therefore need to consider not only expanding MR1T cells but qualitatively shaping their differentiation state toward phenotypes that are most compatible with antimycobacterial protection.

Despite growing enthusiasm, several key questions remain. It is still unclear which MR1T subsets are most protective in TB, how durable vaccine-induced MR1T responses can be, and whether MR1T-targeted strategies can synergize with classical CD4 T-cell immunity, rather than compete with it. Addressing these gaps will require integrated human, non-human primate, and mechanistic studies that combine MR1 tetramer technologies, single-cell profiling, and experimental vaccination models. If these challenges can be met, MR1T cells represent a compelling new axis for TB vaccine innovation—one that leverages donor-unrestricted recognition, rapid effector function, and mucosal enrichment to complement and potentially enhance conventional TB vaccine strategies.

## 5. γδ T Cells

The discovery of γδ T cells dates back to the mid-1980s, when researchers first described a novel type of T cell, that unlike αβ T cells, expressed a TCR comprising a γ-chain and a δ-chain [104,105]. These cells have persisted evolutionarily for 450 million years, since the emergence of jawed vertebrates, highlighting their evolutionary importance [18]. γδ T cells arise early during thymic ontogeny and represent the first T-cell lineage to emerge from the thymus [106]. During fetal and early postnatal life, commitment to the γδ lineage precedes αβ T-cell differentiation, producing waves of γδ T cells that seed peripheral tissues and establish long-lived compartments.

Like other DURTs, γδ T cells have both innate and adaptive features. They respond rapidly to infection with the ability for immediate effector functions, yet are capable of clonal expansion, effector differentiation, and memory-like responses. Human γδ T cells comprise functionally distinct subsets, defined by their Vδ-chain expression. In adult peripheral blood mononuclear cells (PBMC), the vast majority of γδ T cells are Vγ9Vδ2 T cells, composing approximately 5% of the total CD3+ population in healthy adults [107]. In contrast, tissue sites are enriched with cells expressing non-Vδ2 delta chains, such as Vδ1 and Vδ3-expressing subsets. Vγ9Vδ2 T cells recognize microbial phosphoantigens generated by isoprenoid biosynthesis pathways, whereas non-Vγ9Vδ2 T cell populations, including Vδ1 T cells, recognize a broader array of stress- and infection-associated ligands [108].

In the context of TB, γδ T cells expand rapidly following mycobacterial exposure, accumulate at sites of disease, and exhibit potent antimicrobial functions including the cytotoxicity, cytokine production, and immunomodulation of other immune compartments [109]. Both Vγ9Vδ2 and non-Vδ2 T cell subsets have been implicated in the control of TB, with circulating Vγ9Vδ2 T cells responding robustly to mycobacterial phosphoantigens, and tissue-associated γδ T cells contributing to local immune surveillance within the lungs. Together, these features place γδ T cells at the interface of early recognition and effector immunity in TB, supporting their emerging role as contributors to protective responses and attractive targets for vaccine and immunomodulatory strategies.

### 5.1. Vγ9Vδ2 γδ T Cells

In adult blood, approximately 90% of γδ T cells express Vδ2, usually paired with Vγ9 expression [110]. These Vγ9Vδ2 cells have been shown to recognize phosphoantigens, non-peptidic phosphorylated intermediates of metabolic pathways, via a unique mechanism. Specifically, phosphoantigens, such as isopentenyl pyrophosphate (IPP) [111] and (E)-4-hydroxy-3-methyl-but-2-enyl pyrophosphate (HMBPP) [112], bind to an intracellular B30.2 domain of butryophilin (BTN), leading to a conformation change in the BTN2/3 extracellular domains, which is directly recognized by Vγ9Vδ2 T cells.

Vγ9Vδ2 T cells represent the best-characterized γδ T cell subset in humans and are positioned to participate in antimycobacterial immunity. Natural phosphoantigens—including prenyl pyrophosphate intermediates of lipid and steroid biosynthesis, as well as phosphorylated nucleotide metabolites—are produced and released in increased amounts by infecting both microbes, including Mtb, and host cells undergoing metabolic reprogramming during infection [111,113,114]. Early studies demonstrated that BCG vaccination and mycobacterial exposure can prime and expand Vγ9Vδ2 T cells, leading to enhanced responsiveness to phosphoantigens and mycobacteria [115,116]. These findings established that Vγ9Vδ2 T cells are not only rapidly reactive but can also acquire memory-like properties following a mycobacterial encounter, which is consistent with having a role in vaccine-induced immunity.

In vitro studies have shown that phosphoantigen-activated Vγ9Vδ2 T cells can inhibit intracellular Mtb growth through perforin- and granulysin-dependent mechanisms and can directly lyse infected macrophages [117]. These cells also produce cytokines such as IFN-γ and TNF in response to Mtb [118], positioning them to both kill infected targets and shape downstream adaptive immunity. During TB, alterations in Vγ9Vδ2 T cell frequency and function have been reported, with impaired cytokine and granulysin production that normalize following successful therapy, consistent with either redistribution to tissues or functional exhaustion during disease [119]. Moreover, preferential activation of distinct Vγ9Vδ2 T cell clonotypes following BCG exposure suggests functional heterogeneity within this compartment, raising the possibility that specific subsets may be most relevant to protective immunity [116].

Because phosphoantigen-reactive Vγ9Vδ2 T cells are largely restricted to primates, non-human primate (NHP) models have been instrumental in moving beyond association toward causality. In macaques, a pharmacologic or vaccine-based expansion of Vγ2Vδ2 T cells (the primate homolog of human Vγ9Vδ2 cells) results in the pulmonary accumulation of these cells and is associated with reduced mycobacterial burden, attenuated lung pathology, and enhanced expression of IFN-γ, perforin, and granulysin [120].

Compellingly, adoptive transfer experiments have provided direct evidence of protective potential. Transfer of ex vivo-expanded Vγ2Vδ2 T cells into Mtb-infected macaques led to improved control of infection, demonstrating that this lineage can mediate antimycobacterial effects in vivo under defined conditions [121]. NHP vaccination studies have further shown that mucosal or phosphoantigen-targeted immunization strategies can induce durable, multifunctional Vγ2Vδ2 T cell responses that localize to the lung and exhibit rapid recall upon mycobacterial challenge [122]. However, not all approaches that expand circulating Vγ9Vδ2 T cells improve TB outcomes [123], underscoring the importance of tissue targeting, differentiation state, and integration with other immune compartments. Recent work demonstrates that peripheral expansion alone may be insufficient without effective recruitment to pulmonary or granulomatous sites and appropriate effector programming [123].

### 5.2. Non-Vγ9Vδ2 γδ T Cells

Although the role of Vγ9Vδ2 T cells in protection from Mtb is better established, there is growing recognition of the important role that non-Vδ2-expressing γδ T cells play. Non-Vγ9Vδ2 T cells possess several distinct characteristics from Vγ9Vδ2 T cells that make them potentially more attractive targets for TB vaccine development. Vγ9Vδ2 T cells emerge from the thymus with effector and cytotoxic functions already established and have a limited TCR repertoire that is highly public [124,125,126]. By contrast, non-Vδ2 expressing T cells display a phenotype that is much more akin to adaptive cytotoxic CD8 T cells, in that antigen exposure drives upregulation of critical transcription factors, such as TBX21, EOMES and PRDM1 [127], resulting in clonal expansions and the development of the cytotoxic effector function. This has been well-established for other infections, including CMV and malaria [127,128,129]. Non-Vδ2-expressing T cells have also been shown to respond to the mycobacterial antigen α-GalCer via CD1d presentation [51]. Together, this suggests that non-Vδ2-expressing γδ T cells, such as Vδ1 T cells, could be shifted through future TB vaccine exposures from a naïve phenotype to a long-lived memory effector phenotype, making them attractive vaccine targets.

Following neonatal BCG vaccination, work by Gela et al. showed that γδ T cells were significantly increased [130]. However, in a separate study, Papadopoulou et al. showed that there were no significant differences in Vγ9Vδ2 frequencies after neonatal BCG vaccination [124]. This would suggest that BCG vaccination in neonates leads to the expansion of non-Vγ9Vδ2-expressing T cells. Given that in early life, the majority of γδ T cells are non-Vδ2 expressing, early-life BCG exposure may provide a crucial window to study the effect of BCG’s effect on non-Vδ2 expressing γδ T cells, which are likely to become tissue resident γδ T cells in later life. The increased tissue-residency of non-Vδ2 expressing γδ T cells also make them particularly attractive potential vaccine targets, as they are enriched in the lungs, the initial point of infection of Mtb.

The enrichment of Vδ1 at barrier sites suggests that they are providing a specific protective function at these sites [131,132]. Specifically, during pulmonary TB, lung-resident Vδ1-expressing cells have been shown to be skewed towards populations of highly clonally expanded effector cells [16]. These expanded clonotypes were highly localized to specific lung sections, suggesting that they occur as a result of direct antigen exposures. Work by Chowdhury et al. found that in adolescents with controlled/persistent Mtb-infection, there was an expansion of NK-like CD8^+^ γδ T cells, suggesting that they may play a role in the control of Mtb infection [133]. Intriguingly, these expanded γδ T cells were Mtb reactive, but not phosphoantigen reactive, suggesting that they are more likely to be non-Vδ2 expressing γδ T cells.

## 6. Summary and Future Directions

The global burden of both TB disease and TB mortality, despite the nearly 90% BCG vaccination rate and the relative lack of early-stage candidates in the vaccine pipeline [134], highlights the urgent need for novel vaccines. Despite more than a century of reliance on BCG and decades of efforts to optimize peptide-based subunit vaccines, durable and sterilizing protection against pulmonary TB has not been achieved. The historical emphasis on classical CD4^+^ T-cell immunity has yielded critical insights into host defense, but has also revealed clear limitations: IFN-γ-producing CD4 responses, while necessary, may not be sufficient. Naturally acquired infection does not reliably protect against reinfection [10] and vaccine-elicited CD4 responses have, to date, provided limited clinical efficacy [9]. Important roles have been described for conventional CD8^+^ T cells, B cells, and innate immune populations in antimycobacterial defense, highlighting that protective immunity to Mtb infection is multifaceted and extends well beyond CD4^+^ T cells alone. These pathways contribute to the cytotoxic clearance of infected cells, antibody-mediated and myeloid-modulating functions, and early containment of infection, respectively. Yet, many of these responses remain shaped by classical restriction systems or display variable population coverage and durability when leveraged in isolation. Together, these realities compel a broader conceptual framework for TB immunity—one that extends beyond conventional MHC-restricted paradigms.

Donor unrestricted T cells offer such a framework. Across CD1-restricted, HLA-E-restricted, MR1-restricted, and γδ T-cell compartments, several unifying principles emerge. DURTs demonstrate some of or all attributes that position DURTs as attractive targets for TB vaccines. These attributes include: positioning at the mucosal front line, enrichment in the airways and lung parenchyma where Mtb first establishes infection, rapid response to infected cells, deployment of cytotoxic and macrophage-activating effector programs, and recognition of highly conserved non-peptide ligands that are less vulnerable to antigenic variation and population HLA diversity. These populations have been shown to be shaped by BCG vaccination and dynamically regulated across the spectrum of Mtb infection from controlled infection to disease. Experimental models further demonstrate that selected DURT subsets can directly restrict mycobacterial growth, traffic to granulomas, and in some contexts mediate measurable protection.

Rather than replacing conventional T-cell immunity, DURTs may be best viewed as a complementary axis of defense—one that bridges innate immediacy with adaptive specificity. Their ability to act early, amplify local immunity, and sculpt downstream responses positions them as potential gatekeepers of infection outcome. In this regard, DURTs may influence whether inhaled bacilli are eliminated, contained, or permitted to establish long-term niches that eventually culminate in clinical disease. Harnessing this biology could fundamentally shift TB vaccine design towards the inclusion of targeting DURT to achieve programming of rapid, tissue-localized, multi-effector immunity. MR1 ligands, CD1-presented lipids, and phosphoantigen-BTN pathways provide chemically defined and evolutionarily conserved targets that could be incorporated into rationally engineered vaccines. Mucosal delivery platforms, recombinant BCG strains, and vectored or nanoparticle-based systems offer opportunities to preferentially expand and imprint lung-resident DURT populations. Early-life vaccination represents an especially compelling frontier, as DURTs are developmentally prominent, highly plastic, and rapidly shaped by microbial exposure at a time when conventional adaptive immunity remains immature. Strategically engaging these compartments during this window could yield durable tissue immunity that is not readily achieved later in life.

Translating DURT biology into vaccine design will require platform- and route-specific strategies that align with their unique antigen recognition and tissue distribution. Live attenuated vaccines currently provide the strongest evidence for engaging DURT responses in vivo, as exemplified by intravenous BCG, which induces robust expansion and functional programming of multiple unconventional T-cell populations, including MR1T and γδ T cells, in non-human primates. These approaches likely benefit from sustained antigen presentation, access to intracellular antigen-processing pathways, and broad engagement of innate immune signals. In contrast, subunit and mRNA-based platforms may require the incorporation of defined non-peptide ligands—such as MR1 agonists or CD1-presented lipids—together with appropriate adjuvants to effectively prime DURT responses. The delivery route is also likely critical: mucosal vaccination may preferentially engage tissue-resident DURTs in the lung, whereas systemic delivery may be less effective in targeting these compartments. Despite these emerging principles, direct comparative data across platforms and DURT subsets remain limited, and it is not yet clear which combinations of antigen, adjuvant, and delivery strategy will most effectively induce durable and protective DURT-mediated immunity in humans.

At the same time, enthusiasm must be matched with rigor. Key questions remain unresolved: which DURT subsets and differentiation states are most protective; how DURT functions intersect with immunopathology; whether or not DURTs can be durably programmed by vaccination; and how best to integrate DURT-targeted strategies with classical T-cell and antibody-mediated approaches. Progress will depend on coordinated human cohort studies, mechanistic in vivo models, and system-level profiling to define actionable correlates of protection. Advances in tetramer technology, single-cell multi-omics, spatial biology, and controlled human or NHP vaccination models will be central to this effort.

Across DURT subsets, several common themes emerge. Many are enriched at sites of Mtb infection, exhibit rapid effector responses, and recognize conserved non-peptide antigens, supporting their potential relevance in early immune control. However, key differences likely shape their functional roles. For example, MR1T and γδ T cells may be particularly important in early or mucosal responses, whereas CD1- and HLA-E-restricted T cells may contribute more prominently to antigen-specific cytotoxicity and immune modulation. Despite these distinctions, there is currently limited evidence defining whether these populations act synergistically or redundantly in vivo, and whether targeting one subset over another would provide a superior vaccine benefit. As such, a unifying model of DURT coordination in TB remains to be established.

## 7. Conclusions

In sum, DURTs reveal a parallel immunological architecture for sensing and responding to Mtb—one shaped by millions of years of host–microbe co-evolution and that is uniquely suited to the metabolic and mucosal ecology of TB. Leveraging this architecture does not guarantee success, but it offers a rational path beyond the constraints that have historically limited TB vaccines. As the field moves toward next-generation strategies, integrating DURTs into the core logic of vaccine design may prove essential for finally achieving durable, population-wide protection against one of humanity’s oldest pathogens.

## Data Availability

No new data was created.
